# Endoscopic Knot‐Tying of the Nasal Cavity and Skull Base Without Special Instruments

**DOI:** 10.1002/oto2.70137

**Published:** 2025-06-19

**Authors:** Tomotaka Hemmi, Kazuhiro Omura, Kazuhiro Nomura, Teppei Takeda, Satoshi Aoki, Teru Ebihara

**Affiliations:** ^1^ Department of Otolaryngology Japanese Red Cross Ishinomaki Hospital Ishinomaki Miyagi Japan; ^2^ Department of Otolaryngology Tohoku Kosai Hospital Sendai Miyagi Japan; ^3^ Department of Otolaryngology, Head and Neck Surgery Tohoku University School of Medicine Sendai Japan; ^4^ Department of Otolaryngology The Jikei University School of Medicine Minato‐Ku Tokyo Japan; ^5^ Department of Otolaryngology Dokkyo Medical University Saitama Medical Center Koshigaya Saitama Japan

**Keywords:** endoscopic surgery, knot‐tying, nasal cavity, skull base, wound closure

## Abstract

This report introduces an endoscopic knot‐tying technique for a wide range of procedures, such as nasal mucosa suturing and dura mater reconstruction, without the need for specialized instruments. The technique utilizes basic tools like a needle holder, sutures, and bayonet‐shaped nasal forceps. The surgeon ties a surgeon's knot, guided by an endoscope, and pulls the suture with forceps to ensure proper tension. The method is effective in various surgeries, from septoplasty to skull base procedures, and has shown no adverse events in 137 patients. Its advantages include simplicity, no need for specialized tools, and ease of use in different facilities. The technique has the potential to advance endoscopic surgery, providing an efficient solution for diverse surgical applications.

Endoscopic surgery is advancing rapidly with the development of surgical assistance instruments. However, expanding the range of techniques that can be performed with traditional surgical instruments is equally important. Additionally, disparities in healthcare access between developed and emerging countries may limit the availability of specialized surgical tools. This report details an endoscopic knot‐tying technique we developed for a wide range of applications, including nasal mucosa suturing and dura mater reconstruction, without requiring specialized instruments.

## Methods

The essential tools for this technique are a needle holder, sutures, and bayonet‐shaped nasal forceps (Supplemental Video 1). The needle holder should be suitable for manipulation within the nasal cavity. We prefer polyglactin 910 sutures (5‐0 Coated Vicryl with a TF needle, 13 mm, 1/2c; Ethicon Inc.), with gentamicin ointment (Gentamicin Sulfate Ointment 0.1%, IWAKI; Iwaki Seiyaku Co., Ltd.) applied to enhance smoothness.

**Figure 1:**
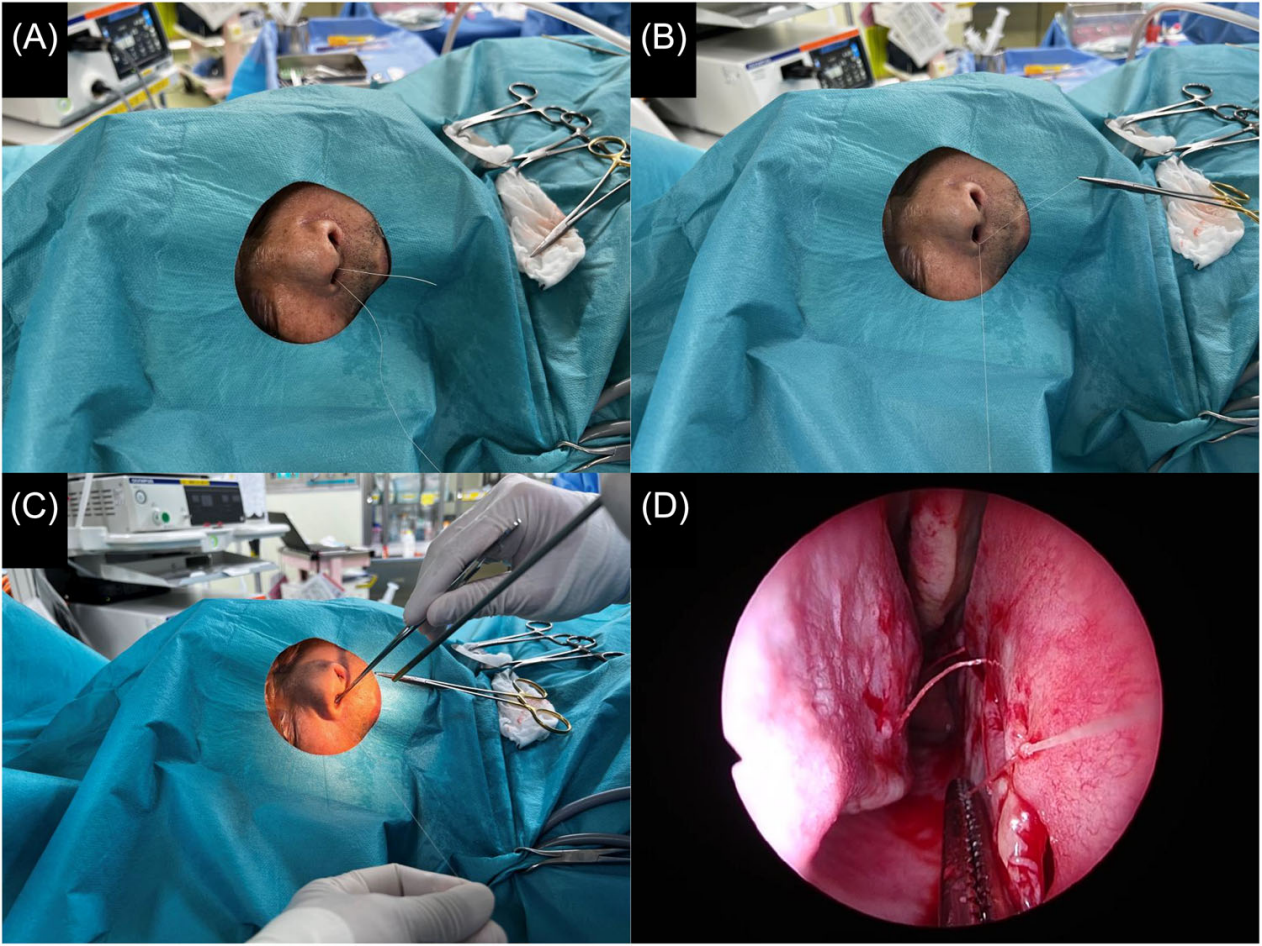
The overview of the procedure with an assistant.

To begin, the suture extends 4 to 5 cm from the nasal cavity (Figure 1A). For posterior nasal cavity or dura mater knot‐tying, the suture should remain outside longer. The surgeon ties the first knot of the surgeon's knot (Figure 1B), and the assistant holds the needle side while the surgeon positions the needle holder to the left of the patient's nostril, pinching the suture's back end. The surgeon uses the left hand to hold the endoscope and the right hand to hold the forceps. While the assistant pulls the suture, the surgeon grasps the posterior end with forceps and advances the suture, guiding the knot to the wound site via the endoscope (Figure 1C). For optimal results, the forceps should pull opposite to the assistant's tension (Figure 1D). The second knot of the surgeon's knot is tied, completing the suture.

**Figure 2:**
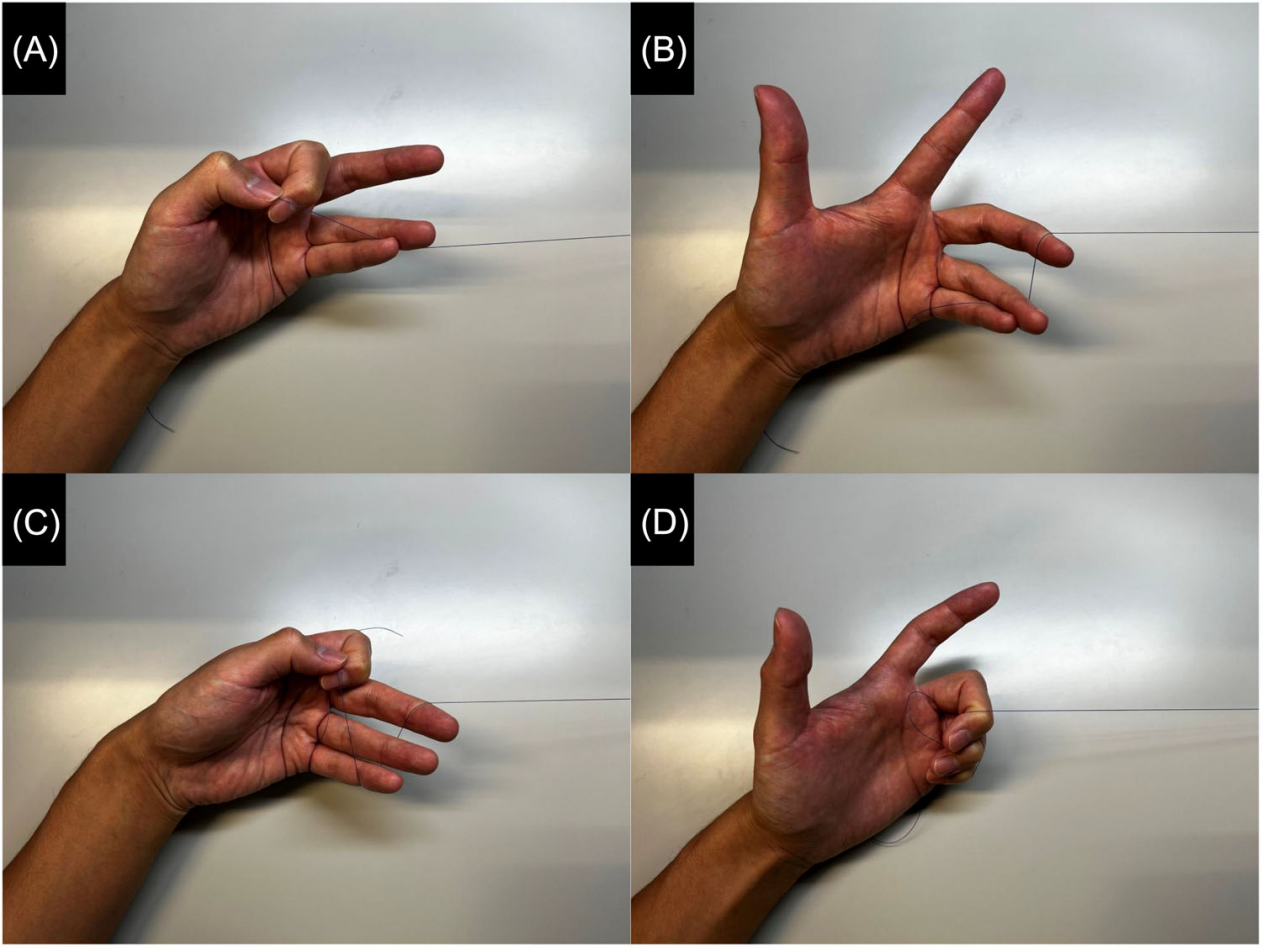
Original method and modified method without an assistant.

**Figure 3:**
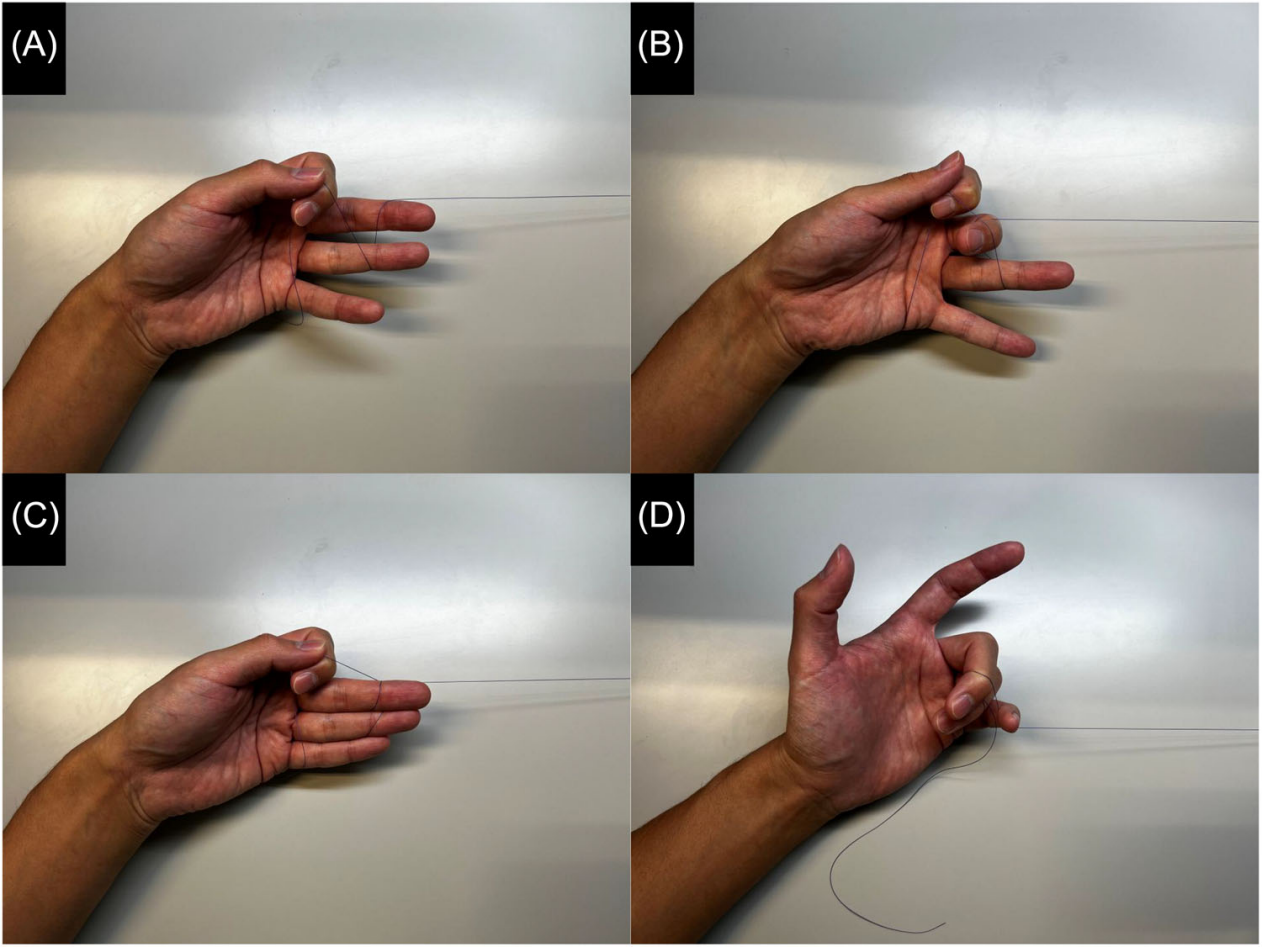
Other modified method without an assistant.

When performed independently, the technique is similar, except that the surgeon's left hand simultaneously pulls the suture while holding the endoscope. The endoscope is gripped with the thumb and index finger, and the suture is held with the remaining fingers. In our original method, the surgeon uses the ring and little fingers to hold the suture and pulls it with the middle finger (Figures 2A and B). A modification by others involves using only the distal interphalangeal joints of the little finger (Figures 2C and D). If more tension is needed, the surgeon may use the middle and ring fingers to hold the suture and the little finger to pull it (Figures 3A‐D). Regardless of technique, the suture should be held as close to the patient's face as possible, with adjustments made so the wound remains visible through the endoscope.

This study was approved by the Institutional Review Board (kkrtohoku‐202312otor_S1‐1_01) and adhered to the Helsinki Declaration of 1975, as revised in 1983.

## Results

A total of 137 patients underwent procedures between January 2023 and August 2023 using this technique (Supplemental Table S1, available online). The authors (T.H., K.N.) performed suturing on mucosal incisions made during septoplasty, inferior turbinate reduction,^1^ and endoscopic modified medial maxillectomy.^2^ Additionally, the authors (K.O., T.T., S.A., and T.E.) used this technique for dura mater reconstruction after anterior skull base surgery. No adverse events, such as suture failure or wound infection, cerebrospinal fluid leakage, were observed.

## Discussion

The use of specialized instruments for nasal suturing is common, and some surgeons have developed techniques for deep knot‐tying, like dura mater reconstruction.^3^ However, there are concerns about knot‐tying complexity and potential risks, such as excessive tension. Our method offers several advantages: no need for specialized instruments, ease of use in any facility, no complex knot‐tying techniques, and the ability to apply appropriate tension. This method is effective for various procedures, from nasal mucosa suturing to dura mater reconstruction.^1^, ^4^ Furthermore, sutures tied using this technique spontaneously drop within 2 to 3 months,^5^ which is consistent with the absorption period indicated in the official product documentation, and we consider it sufficient for wound healing.

If an assistant is available, the procedure becomes easier with their help in pulling the suture. However, performing the procedure independently allows the surgeon to receive immediate feedback on the tension applied, improving skill and control. We outlined three ways to hold the suture, but surgeons should choose the method that best suits them. As long as a point of support and a point of force are established using the middle, ring, and little fingers, any technique can be effective. Surgeons should continue practicing their preferred method.

For more complex procedures, such as dura mater reconstruction at the middle skull base, it may be worth considering for use of a needle holder that allows for more delicate operations such as EASY KONT K.O. GRIP (DAIYA SEIKI Co. Ltd.).

## Conclusion

We described an endoscopic knot‐tying technique suitable for a wide range of procedures, from nasal mucosa suturing to dura mater reconstruction, without relying on specialized instruments. Endoscopic nasal surgery is rapidly evolving, and effective wound closure remains a key aspect of successful procedures. We hope this method contributes to the further development of endoscopic surgery.

## Author Contributions

Tomotaka Hemmi and Kazuhiro Omura contributed equally to this study. Kazuhiro Omura devised the original technique (Figures 2A and B), Teppei Takeda, Satoshi Aoki, and Teru Ebihara modified the original technique (Figures 2C and D), Tomotaka Hemmi and Kazuhiro Nomura devised the final form (Figures 3A‐D).

## Disclosures

### Competing interests

None.

### Funding source

None.

## Supporting information


**Supporting Information**.

**Supporting Information**.
