# Supplementary material for: Endoscopic Knot‐Tying of the Nasal Cavity and Skull Base Without Special Instruments
Source: OTO Open. 2025 Jun 19;9(2):e70137. doi: 10.1002/oto2.70137 (PMC12177785; doi:10.1002/oto2.70137)
Supplement: Supplementary file 1 — Supporting Information. [file OTO2-9-e70137-s002.docx]

| Item | Value |
| --- | --- |
| Patients | 137 |
| Operative procedure (number of patients) | |
| Inferior turbinate reduction | 99 |
| Deviatomy with Killian incision | 78 |
| EMMM | 12 |
| Anterior skull base surgery | 11 |
| Suture site (number of patients) | |
| Lateral nasal wall | 111 |
| Nasal septum | 78 |
| Skull base | 11 |

**Table S1.**

Title: Details of the patient group.

Description: Operative procedures and suture site are indicated with duplicates. EMMM: endoscopic modified medial maxillectomy.
